# The Role of Personal Values in Learning Approaches and Student Achievements

**DOI:** 10.3390/bs11070102

**Published:** 2021-07-16

**Authors:** Kelum A. A. Gamage, D. M. S. C. P. K. Dehideniya, Sakunthala Y. Ekanayake

**Affiliations:** 1 James Watt School of Engineering, University of Glasgow, Glasgow G12 8QQ, UK; 2 Department of Education, University of Peradeniya, Peradeniya 20400, Sri Lanka; scpkdehideniya@gmail.com (D.M.S.C.P.K.D.); sakuyatigammana@gmail.com (S.Y.E.)

**Keywords:** personal values, learning approaches, student behaviours, attitudes

## Abstract

Personal values play a significant role when adopting learning approaches by individuals during their studies. Particularly in higher education, these values significantly influence the character that individuals play within their learning community and ultimately influence their academic achievements. The purpose of this paper is to investigate personal values in their choice of learning approaches and, subsequently, how it impacts one’s academic achievements. It also investigates the importance of developing an individual’s personal values as a part of their wider studies, while aligning these with graduate attributes and balancing them with knowledge and skills, to produce successful graduates in a society.

## 1. Introduction

Values are the fundamental beliefs, behaviours and attitudes that have been approved and accepted as what is good by society for a long time. In the most general sense, they are considered as the virtues that a person holds in his or her life. However, philosophers, researchers, practitioners and many others have defined and addressed values from different perspectives concerning the respective discipline or contexts. Generally, values are viewed as inner realities of an individual that are reflected through habits, behaviours, beliefs, expectations and relationships. Values lay the foundation for an individual’s pattern of thinking and way of acting. They play a vital role in how one makes decisions, choices and builds perceptions and attitudes. Additionally, various studies on personal values have shown that they often guide decision making in all aspects of life such as career, religion, social circles and self-identity [1]. Another aspect of personal values is that they can be viewed as desirable motivational goals and interests of an individual or the guiding principles in life. In addition, they have been seen as the non-existent mental entities and as the outcomes of mental development. Consequently, values can be seen as the perceptions of psychological expressions or frame of mind. Moreover, they are consequential issues that manifest the personality of an individual. Hence, the realisation of personal values by the self is crucial and determines the growth and the existence of the self in any situation. Conversely, understanding another person’s respected values is also important.

As a consequence of the constant transformation of society in terms of culture, economics and politics, value and value systems have been unusually changed and distorted. In favour of the same idea, Daniela et al. [2] justify this by arguing, “with modernity it is normal that personal value systems support changes to harmonize itself to current requirements”. Initially, some of the personal values may be determined by birth and later greatly influenced and molded by education, experiences, society, culture and many other factors. According to Matthews et al. [3], an alteration in lifestyle, cultural environment and economic circumstances, individually or a combination of these factors, can cause values to change. Personal value systems can be viewed as a relatively permanent framework that exists within an individual which decides what is good or bad for himself or herself and his or her companions. In addition, it shapes and influences the general nature of an individual’s behaviour. Researchers have found that personal values developed early in life may be resistant to change and may be derived from those of particular groups or systems, such as culture, religion and political party. However, personal values are not universal. Genetically inherited features and external factors including education may determine one’s personal values. Essentially, the antecedents of values are culturally embedded in society and its institutions [3] and are socially determined [2]. Although a personal value is an internal phenomenon, the motivating force to adopt the value is seen as emanating from a diverse range of external sources [4]. As values directly influence one’s entire lifestyle, a discussion of values and value systems, their place within changing socioeconomic contexts and how they affect individuals and society has universal relevance.

The value systems of a society always determine human activity in social life, education and professional life. Values are seen as a key component of organisational culture and are repeatedly defined as the principles accountable for the successful management of the organization [5]. Arambewela and Hall [6] support the same issue, stating [7]: personal values have long been considered an important variable in understanding consumer behaviour and decision making. As a result, the interest in knowing the drivers behind consumer attitudes and behaviour has encouraged marketing researchers to investigate human values [8] (Anana and Nique, 2014). Hence, many researchers have emphasised the need and advantage of studying the impact of personal values on the sustainable existence of an organisation.

Investigating the influence of values on assessments made by people on their career choices is another trending research area. In general, professions such as teaching, medicine and nursing are strongly attached and influenced by values. The results of a research study by Anana and Nique [8] has concluded that students choosing some careers are more typical, based on their values than others, and that some values are more typical of some careers than others. Thus, personal values have been taken as the main focus in the research in a variety of fields and academic disciplines ([3] cites Feather, 1975). In this regard, the need for identifying different scopes of human values is a timely requirement. Since professional values are also shaped and influenced by personal values, indeed a discussion on personal values can be regarded as an issue that unchanged over time.

There has been a growing concern over the erosion of values among youth during the past few years, and it is continually progressing. At the same time, the need for facilitating value development has become the greatest challenge ahead in the field of education. The effects of the value given to material comforts, money, fame and success are prominently reflected through the present younger generation. Hence, there is a considerable emphasis in this new century on the development of values: tolerance, social justice, open-mindedness, empathy and deep respect for others. Since realizing values and adopting and displaying them as one’s personality is closely associated with education, values education is given a greater emphasis today to ensure the continuity of societies. The functions of education in molding student’s moral, spiritual and sociocultural life are some of the areas that have received renewed attention in the recent past. In addition, they have long been considered important variables in understanding student behaviours, attitudes and achievements. Consequently, the outcomes of Branson [4] provide an insight into the benefits of value-based studies in educational management and administration. The realization of value can offer assistance in organizing the learning process by explaining and understanding students’ reactions to various situations and tailoring and evaluating the learning experience. Though students’ learning takes place within the self, it is not an isolated process. Research has confirmed that learning is affected by a variety of internal and external factors. Researchers have observed variations in students’ learning approaches, and furthermore, they have found qualitative differences in learning outcomes that were related to the approaches taken (Matthews et al. [3] refer Marton and Saljo [9]). Accordingly, if the personal values deal with the behaviour of a person, learning may also have influenced by personal values. Based on that assumption, a number of researchers study the composition and structure of students’ learning approaches and personal values [10] and their interconnections have been observed in various contexts. Research in this area confirms that values are related to different approaches to learning and they may change according to the circumstances. Considering the students’ behaviour in different academic situations, researchers have categorized the learning approaches into different groups. Furthermore, researchers have attempted to build up the connections between learning approaches and specific personal values. For example, as referred by Matthews et al. [3] and Tarabashkina [10], personal values such as achievement and power were related to the achieving approach, security and tradition values to the surface approach, and self-direction and universalism to the deep learning approach. In addition, this relationship was confirmed by a number of studies with some variations.

The values occupy a pre-eminent position on the agenda of researchers in education and many other domains as they impact behaviour, attitudes, expectations and all the other personal characteristics and constructs. Hence, this paper seeks to contribute by reviewing the available literature on the role of personal values concerning learning approaches and student achievements. The review centres on the following given objectives.

- To investigate the role of students’’ personal values in their choice of learning approaches;
- To investigate the impact of personal values on one’s academic achievement;
- To investigate the importance of developing individual’s personal values as a part of their academic life;
- To investigate how one’s personal values shape the learning community around that person and vice versa.

## 2. Background Literature

The background literature aims to synthesise the most relevant research outcomes for the main topic of study under the four main areas: personal values, personal value theories, value education and learning approaches. The concept of personal values is quite closely connected with value theories and value education. In reality, they are inseparable and cannot be treated separately since they are branches of the same root. About the very same idea, to define, describe and to understand personal values, several frameworks have been used by the researchers. Thus, the historical evolution of personal values can be identified through the presented frameworks. Moreover, as the literature suggests, through empirical evidence, there exists a relationship between personal values and students’ learning. Hence, uncovering the background literature through the above four areas are important for the total comprehension of the reader.

### 2.1. Personal Values

As a whole, personal values significantly influence all aspects of one’s life. It is also obvious that values contribute to the building of one’s personal and social identity. Understanding the concept of personal values is indeed a complex process. Over the past years, it has been viewed diversely analysing from the individual level and up to organisational, institutional, social and cultural levels [2,11,12], resulting in several definitions addressing different scopes.

Personal values or individual values are the values to which an individual is committed and which influences his behaviour [13]. As Ledden et al. [14] view, value perceptions are the result of a cognitive trade-off between benefits and sacrifices. According to Rokeach [7], a value is a long term belief that a certain path or purpose of existence is preferable from the social and personal point of view over another one in the opposite [2]. Furthermore, values can be referred to as interests, desires, goals, needs and standards of preference [3]. (Ros [15] supports the same concept and states, “a value is a desirable state, object, goal or behaviour transcending specific situations and applied as normative standard to judge and to choose among alternative modes of behaviour” [2,15].

Moving a little from the basic components mentioned in the previous definitions, Anana and Nique [8] say that a value is a reference people use to judge themselves and others or to influence the values, attitudes and actions of other people, such as children. People who use the features obtained through the sense organs in defining other beings can benefit from the impressions they have emotionally in attributing importance to that being and appraising it [16]. These emotion-based impressions are generally called “values”. Another definition suggests that values are systematic and, to some extent, precise ideas that ensure the interaction of an individual with the environment [17]). Regarding the concept of personal values, Mashlah [5] and Daniela et al. (2013) [2] refer to Schwartz and Bilsky’s [18] and Schwartz’s [19] definition of values as a combination of five main features: values are (a) concepts or beliefs (b) about desirable end states or behaviours (c) that transcend specific situations, (d) guide the selection or evaluation of behaviour and events and (e) are ordered by relative importance. Analytical observation on the definitions of values shows that they are more or less diverse in meanings. Basically, terms such as interests, beliefs, desires and behaviour have been used in common in definitions. However, when focused, it is evident that values have been defined as concerning the cognitive, affective and behavioural aspects of an individual.

In contrast to the above, Ledden [14] calls attention to another two important points referring to the relevant literature. Firstly, based on the literary evidence, researchers state that value’s loose definition and the diverse nomenclature used by authors have collectively led to some authors using the term value interchangeably with concepts such as satisfaction, quality and values, particularly with the personal values that guide human behaviour such as beliefs of right and wrong. Secondly, as Ledden states [14], despite the consensus on terms and definition of values, the literature evidences some confusion in differentiating between the concept of the value and the notion of values. The argument is supported with an important distinction between value (singular) and values (plural) marked by Holbrook [20], defining the former as a preference judgment and the latter as the criteria by which people make such preference judgments; thus, value is related to, but distinct from, the concept of values. In a general sense, this diversity possibly emerges through the variations in values referred to in different research domains.

Adhering to the research outcomes, Branson et al. [4] discusses the adoption of values by a person. As explained by values theory, a person’s values are dependent upon his or her consciousness and those values are unique to the person. Research shows that people do not learn values, but rather, they unconsciously adopt values. For example, values are adopted subliminally rather than being consciously selected and deliberately adopted by the individual [4]. Each person sees a unique and specific view of their world due to the influence of his or her conscious perceptions.

Turning to the historical traces of debates and discussions on personal values, values are abstract concepts that have been studied since ancient times [21] and can be traced back to the lessons from Aristotle, Plato and Socrates [15]. Evidently, research into value education has been carried out for almost centuries [3] and continues today. As a result, several definitions and models have been suggested and empirically studied over the past years. Despite the key components and focuses, several models are found frequently cited in the research literature. As Hanel, Litzellachner and Maio [11] suggested, the following are at the forefront of all the other individual value models.

- Spranger’s (1921)—Model of types of people;
- Rokeach’s (1973)—Instrumental and terminal values;
- Schwartz’s—The Schwartz (1992) theory of basic human values;
- Gouvela’s (2013)—Functional theory of values.

Value theories focused on values at the individual level as well values can also be described on a cultural level. As Hanel, Litzellachner and Maio [11] refer, three prominent approaches of this type were proposed by Inglehart, Hofstede and Schwartz [19].

Particularly for this article, Schwartz’s model of human values is adopted as the fundamental theory to discuss the issues highlighted in the objectives since it has been referred to as the theoretical ground of a number of recent research studies on personal values in a variety of contexts. Specifically in the studies which examined how the basic values relate to various attitudes, opinions, behaviours, personalities and background characteristics. In addition, it has been used in hundreds of studies that assessed value transmission and development in an individual from childhood to adolescence and value change over time. Moreover, the theory itself concerns the basic values that people in all cultures recognize [19]. Thus, it can be accepted as universally applicable without any bias. Additionally, considering the very diversity of meanings of the construct of values, the sociopsychological aspects of values are focused on throughout this article.

### 2.2. Personal Value Theories—Schwartz Theory of Human Values

Values can range from the simplest forms, such as punctuality and kindness, to pretentious forms such as self-direction, universalism and conformity. Over the past years, various value models have been proposed and empirically supported [11]. All of them have often defined human values as abstract ideals that guide people’s behaviour and are crucial for explaining social and personal organizations and tracing their changes due to various factors. Among the different value models that have been suggested, the Schwartz [18] theory of basic human values is found frequently cited in the literature.

As Schwartz described, there are six main features of values according to the theory [18,19]: values are beliefs linked inextricably to affect, values refer to desirable goals that motivate action, values transcend specific actions and situations, values serve as standards, values are ordered by importance relative to one another and the relative importance of multiple values are guides to action. These six features apply to all values. Furthermore, theory distinguishes ten basic values (value types) which encompass the range of motivationally distinct values recognized across cultures. These values are likely to be universal because they are grounded in universal requirements of human existence. However, they differ in their motivational content. The definitions of the ten values in terms of the broad goals they express in Table 1.

**Figure 1:**
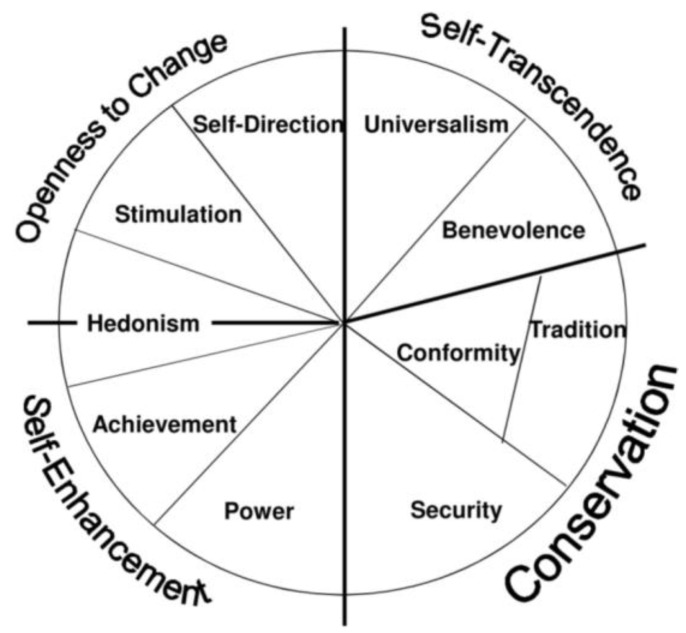
Theoretical model of relations among ten motivational types of values.

He presented the structural model of basic values which takes the form of a circle. Complementary values, i.e., values that are similar to motivational content, are located side by side on this circle while competing values are located at opposing sides [18,19]. The closer any two values in either direction around the circle, the more similar their underlying motivations; the more distant, the more antagonistic their motivations [19]. It seems that the whole set of ten values relates to each other closely or distantly and by that mean they may interrelate with any other variable such as behaviour, attitude, age, etc. (Figure 1).

### 2.3. Value Education

The concept of values has been defined differently in the literature depending on the contexts and the situations. However, along with the rapid changes in the world, the concepts of values and value education have gained renewed attention due to the increased social immorality [21]. Value education can address different forms and definitions. In religious senses, it is most possibly defined as moral and spiritual development. To sociological concepts, it can be termed as the part of socialisation and personality development or the transmission of cultural elements. In the dimension of education, it is addressed through citizenship education. However, in the most general sense, value education stresses the process by which people develop moral values and transfer them through factors such as social relationships, religion and education.

The values, attitudes and personal qualities of young people and the role of the school in spiritual, moral, social and cultural development have received renewed attention in recent years [21,22,23]. As education is a personality-building process [24], school education is challenged by preparing students to face the complexities of future life. Rapidly changing socioeconomic structures and their consequences in terms of patterns of work, family life and social relationships requires an educational response. In that context, experts have recognised the 21st century school curriculum as the most influential mode of transferring values to the younger generation other than the family and other immediate social units. Sahin [16] suggests that implicit or planned values education in schools plays an active role in transferring values from society to society. By its definition, value education refers to those pedagogies that educators use to create enriching learning experiences for students and addresses issues related to character formation [25] and moral development. Moral values are the values that make individuals distinguish between what is good or bad and right or wrong and simply it gives the ideas about the good personal and social life. Halstead and Tylor [21] refer to a discussion document on Spiritual and Moral Development and highlight that the moral values that school should promote are telling the truth, keeping promises, respecting the rights and property of others, acting considerately towards others, helping those less fortunate and weaker than ourselves, taking personal responsibility for one’s actions and self-discipline. Moreover, schools reject bullying, cheating, deceit, cruelty, irresponsibility and dishonesty.

Sahin [16] has identified the four main characteristics of values education as:

- To raise individuals’ awareness of universal (ethical), cultural values, and their importance;
- To relate democratic attitudes and tolerance to multiculturalism;
- To evaluate all values with the criteria of improving people’s living conditions and facilities;
- To turn life into knowledge and/or knowledge into life considering concrete problems related to ethical values.

Sahin [16] views the main purpose of values education as to make values permanent behaviours in students. Providing students with the knowledge and insight into values and beliefs that enables them to reflect on their experience in a way that develop their spiritual awareness and self-knowledge, teaches them the principles which distinguish right from wrong and teaches students to appreciate their cultural traditions and the diversity and richness of other cultures are among the basic functional aspects of value education provided through the school education [21]. Accordingly, the particular theme of value education is directly related to inculcating moral values in students, and it can be identified as another phase of personal value development since the same aspects are named and described in personal value models and frameworks in more or less similar terms. For example, the features that institutions wish to promote through moral or value education are discussed in the ten basic values in Schwartz theory of basic values under the themes of conformity, benevolence, tradition, security and universalism. As Schwartz [19] views, benevolence and conformity values both promote cooperative and supportive social relations and both values may motivate the same helpful act, separately or together. Traditional values imply one’s affection towards religious beliefs and respect for tradition and customs while security values inspire one’s need for safety and harmony. Hence, through value education, it develops values such as conformity, security, universalism and benevolence.

In developing values in individuals, it is widely recognised that schools are not the only nor are they the greatest influence on the values, attitudes and personal qualities of young people, but parents, communities and other agencies are also influential [21]. The early-stage value development through the family, neighbours, practice of religion, culture and nursery forms the foundation for the personal values system that one holds. It can be further sharpened through the formal and informal educational and cultural practices in the school or any other institution.

### 2.4. Learning Approaches

Approaches to learning mainly focus on how children engage in learning referring to the use of skills and behaviours. In addition, they are discussed incorporating emotional, behavioural and cognitive domains. Learning is a process of changing behaviour through experiences and is relatively a permanent product. Hence, it is important to understand student learning approaches to improve and maintain the quality of the learning experience. Beyaztas and Senemoglu [26] define learning approaches in terms of how a learner’s intentions, behaviours and study habits change according to their perception of a learning task to the context which the learner regards.

According to Lietz and Matthews [27] two major perspectives have guided theory and research into student learning: The first is The Self-Regulated Learning (SRL) rooted in North America, and the second is The Students’ Approaches to Learning (SAL) that is prominent in Europe and Australia/Southeast Asia. In parallel to that, Matthews et al. (2007) [3] cite Biggs’ [28] findings on Asian student learning approaches, and according to it, learning is based on two types: the Information Processing Approach and the Contextually and Experientially Based Learning Approach. The above findings specifically refer to the geographical region and it is reasonable to pose the argument that the variation patterns in learning approaches are existing to the sociogeographical factors such as country, region and culture.

Biggs [29,30] specified three distinct approaches (see Table 2) to learning namely, The Surface, The Deep and The Achieving approaches to learning [3,27]. In addition, each approach is composed of a motivation that directed learning and a strategy for the implementation of the learning approach [3].

Li’s [32] perspective on student learning approaches is quite different from the above and states that students are smart in different ways and have different learning approaches. According to Na Li, the two major perspectives of learning are the constructivist and student-centred learning approaches: Inquiry-based learning, Problem-based learning, the Situated and embodied cognition model, Self-regulated learning and Cognitive apprenticeship model and Technology-enhanced learning approaches.

Research into learning approaches has focused on studying the impact of background factors such as gender, sociocultural backgrounds, discipline area, personal values and the learning culture of students. As highlighted by Lietz and Matthews [27], Cano-Garcia [33] has shown that older female students tended to score higher on the deep and achieving approaches to learning than younger male students. In addition, studies of Jones et al. [34] and Smith and Miller [35] reflected strong relationships between learning approaches and academic disciplines. Beyaztas and Senemoglu [26] reveal another dimension of research on learning approaches in relation to the examination on students’ learning and studying behaviour towards exams and exam types. Results of these interventions revealed that students’ learning approaches change according to the examination type they were preparing for and Ramsden [36] has proposed strategic learning approaches for students who have more exam-oriented study behaviours.

Another major area that researchers concentrated is changes in the learning approach over time. A number of cross-sectional and longitudinal studies have investigated changes in learning approaches over time [3,10,37]. Both Lietz and Matthews [27] and Tarabashkina and Lietz [10] refer to the same group of studies that investigated changes in learning approaches over time. As they arranged into the chronological order the earliest, Watkins and Hattie’s [38] study on a sample of undergraduate students found that the longer students had studied, the more they displayed characteristics of the deep approach to learning. Contrary to the results of their first study, Watkins and Hattie’s longitudinal study [39] showed no evidence of students’ deep learning approaches intensifying over time. However, Biggs [29] reported a general decline in the deep approach from the first to final year of study in a sample of undergraduate students in Australia. However, no significant changes were observed for other learning approaches. In the study by Gow and Kember [40], results showed that older students used the deep approach significantly more often than younger students. In addition, students at the beginning of their studies appeared to prefer an achieving approach compared to students who were further advanced in their studies. In addition, the more time that had elapsed since leaving school, the fewer the number of students who displayed characteristics of the surface approach. In another study by Kember [41], it was uncovered that younger students showed a preference for a more superficial approach in a comparison of first, second and third-year students. In contrast to the results of his study in 1990 [40], he found that first-year students showed significantly higher scores on the deep approach to learning than second and third-year students. Zeegers’s [42] study on a class of chemistry students over 30 months has shown a significant decline in the achieving strategy and a significant increase in the surface strategy over the time of the study. For the deep approach, no statistically significant changes emerged over time. Another study carried out by Matthews [3] on the same issue discovered that students’ approaches to learning generally became deeper over time. In contrast, Cano’s [33] study observed a significant decline from junior to senior high school with regards to the deep and surface learning approaches both in boys and girls.

In general, preference for a deep learning approach has emerged as the major concern of all studies, and there is no specific pattern of applying a particular approach for learning among the students. Hence, there may be some other background factors influencing the selection and application as well as the changing of a specific approach to learning. In the point of factors affecting students’ learning approaches, Beyaztas and Senemoglu [26] summarize the 3P model (Presage, Process and Product), and according to it, prior knowledge, abilities, preferred ways of learning, values and expectations, teaching context (including the curriculum) and teaching methods affect the student’s selection.

As revealed through the research studies, approaches to learning are probable to change in response to gender, ability, formal teaching authority, time, personal values [3,27], the requirements of and as an adaptation to new environments, the learning culture and the academic discipline and its nature [10,27]. Additionally, as Beyaztas and Senemoglu [26] state, referring to an early study of Ramsden [36], students’ perception of their teachers and departments also have important effects on their learning approaches. In addition, the curriculum and sociocultural environment also may have an effect on selecting the learning approach. Thus, it can be concluded that students’ preference for learning approach is influenced by several factors and they may be inborn or situational. In other words, learning approached may be a result of a combination of several internal and external factors including personal value traits.

## 3. Methodological Design

This research is based on a systematic review of the literature with a narrative summary that exclusively depended on online databases. The predetermined selection criteria, which are given in Table 3, were applied during the database search screening of the text titles, abstracts and whole texts.

Following the above-mentioned criteria, full texts that were reported within 20 years were purposely selected due to the availability of a limited number of accessible resources to retrieve the literature. In relation to the year of publication, the search action was conducted with the use of online databases. As the main sources of data, Google Scholar, JSTOR and Elsevier were used. The ResearchGate database was also used for the search of resources.

The comprehensive search resources were completed based on a wide range of key terms and phrases including “values”, “personal values”, “learning approaches”, “learning communities” and “learning approaches—academic achievement and value education”. However, similar terms that are often used interchangeably in the literature were also used. In particular, with regards to the concepts of personal values and value education, they have also been searched through the terms “humanistic values”, “soft skills”, “social skills” and “moral education”.

As the search action resulted in a limited number of appropriate and accessible sources, the reference section of the found texts were studied in the search for more relevant texts. After the exclusion of sources that did not satisfy the criteria in Table 3, 38 texts were selected for analysis. The content of the selected resources was studied and analysed in detail. Then, the required data were organized under four main themes following the study objectives.

## 4. Results

### 4.1. Objective 1: To Investigate the Role of Students’ Personal Values in Their Choice of Learning Approaches

In the most general sense, approaches to learning describe what a student does when he/she is learning and why he/she should do it. In other words, it is the way that students perceive and value the learning process and how they behave during the process. As suggested by the aforementioned facts and information, education correlates with personal values. Hence, a considerable number of educational studies have been carried out to examine the composition and structure of personal values and their relationships with learning approaches. Values are considered to be precursors as well as predictors of behaviour [3]. In the same way, studies have proven that a tendency towards certain types of behaviours depends strongly on the structure of one’s values. Conversely, learning can be seen as a type of individual-specific behavioural pattern. In that respect, it is justifiable to accept that there is a relationship between personal values and the learning approaches of students. In addition, the values are believed to be influenced by background factors such as religion, culture, political factors, age and many others. Assuming that they also definitely influence in preference of a student’s learning approach, research into learning approaches has focused on a variety of backgrounds. According to Lietz and Matthews [27], research studies have focused on studying the differences in choice of learning approach and personal values relationships depending on gender, discipline area of study, prior performance and the experiences of students, especially the students who undertake higher education in another country. With regards to personal values, researchers in this context have confirmed that values are correlated with different learning approaches.

The influence of personal values on life goals are better described as follows: “values refer to desirable goals that motivate action” [19]. Wilding and Andrew’s [43] study results of “Life goals, approaches to study and performance in an undergraduate cohort” can be discussed taking that as the ground. According to them, the deep approach and the surface approach are the two main approaches to studying that have been distinguished by several researchers. In addition, an achieving or strategic approach employs either deep or surface strategies, depending on the demands of the task. The research aimed to investigate factors contributing to the choice of the preferred study approach at university and relations between these factors and academic performance. Based on the results, as the researchers state, this study has shown that approaches to study are related to wider attitudes to life or the general life goals and relations were found to be consistent with the deep approach being associated with altruistic life goals and the surface approach being associated with wealth and status life goals. The achieving approach was related to both types of life goal, but more strongly to wealth and status life goals.

The most frequently referred research of Matthews [3] on sojourner students in Australia has found interesting relationships between values and learning approaches. From the three pairs of canonical variables that emerged out of the analysis the first pair of variables illustrated that students with clearly defined value structure had equally well-defined learning motivations and strategies. The second pair of variables showed that students who had low integrity values showed a higher preference for surface or superficial learning. In contrast, the third pair of variables indicated that students who had a lesser emphasis on values associated with the Confucian ethos showed a strong preference for the deep strategy [3].

In the study of “Values and Learning approaches of students at an international University”, Matthews, Lietz and Darmawan [3] relate the ten values postulated by Schwartz et al. [18] to Biggs’ [29] six subscales and the relationships between values and approaches to learning has been estimated by canonical correlation analysis. It has revealed that values can be linked to learning approaches even in a situation where students have left their home countries to undertake tertiary studies in a new social, cultural and educational environment. There, the results have been interpreted to the higher-order values: self-aggrandisement, conservatism, self-directedness and benevolent change, which were initially termed as self-enhancement, self-transcendence, openness to change and conservation, respectively, as proposed by Schwartz [18]. Four distinct pairings between values and learning approaches were established: (a) self-aggrandisement (Achievement and power values) is linked to the achievement learning approach, (b) conservatism (universalism and benevolence values) relates to the surface learning approach, (c) self-directedness (self-direction and stimulation values) is linked to the deep learning approach and (d) benevolent change (conformity, tradition and security values) is related to the learning strategies variables were emerged as the results.

In terms of the main research question, the impact of students’ personal values on learning approaches and changes in them over time of Lietz and Matthews [27] longitudinal study on “The Effects of College Students’ Personal Values on Changes in Learning Approaches” has given mixed results. The three-year study results have shown no changes within students in the deep and surface approaches to learning but a significant decline for the achieving approach, particularly for students who previously experienced a more formal teaching authority. As they described, the students who identified to a greater extent with the achievement, hedonism and security values have demonstrated a higher achieving approach to learning at the start of their higher education. Conversely, but in line with expectations, students who valued having fun and a good time more than other students have displayed fewer characteristics of the achieving approach to learning. However, none of the personal values were found to influence how the achieving approach to learning changed over time. Based on the research outcome they have concluded that, while personal values appear to explain differences in learning approaches at one point in time they do not seem to contribute to explaining changes in learning approaches over time. In that case, as explained in a similar study by Matthews (2007) [3] students are likely to change both their personal values and learning approaches due to the influence of the new environment or it may result to pursue their education.

Parallel to the theme of the above studies, Tarabashkina and Lietz [10] carried out a longitudinal research study on “The impact of values and learning approaches on student achievement: Gender and academic discipline influences” using a cohort of international students who started their three-year Bachelor of Arts or Bachelor of Science degrees in September 2004 at a university in Germany. According to the results, hedonism and achievement were consistently related to the achieving approach over three years, whereas the achievement value probably had a large positive effect on the achieving approach, and hedonism (that is, the tendency to have fun) was negatively related to this approach across all occasions. Hedonism was also consistently and negatively linked to the deep approach throughout all years, whereas self-direction had a positive impact on this approach over a two-year period. Self-direction emerged as a constant predictor of the surface approach, although in the opposite direction to this effect for the deep approach.

Accordingly, the reported literature provides insights that the personal values and learning approaches are two components that occur at the same time with parallel construction. In addition, it establishes the relationship regarding how personal values are linked with different learning approaches and how these interrelationships change over time.

### 4.2. Objective 2: To Investigate the Impact of Personal Values on One’s Academic Achievement

Personal characteristics such as skills, abilities and values, academic adaptability, concern on learning objectives, decision making, innovation and communication are some of the main features of any valid evaluation criteria. When elaborating on the state of personal values in line with its impact on one’s academic achievement, knowledge as a human-specific activity is in direct relation with the way a person through his values perceives the world, the phenomena and events Daniela et al. [2]. The values favoured by different individuals can be more or less equal or different. Similarly, within each unique and specific view of the world, each person attributes different values to the same experience or the same value to different experiences [4]. Accordingly, the existing similarities and differences in values cause much diversity in behaviour. Typically, human beings tend to adapt their values according to the circumstances. In addition, it can be assumed that the values do reflect themselves through all the activities of individuals. Identifying the worth of studying these variations, in addition to exploring the link between values and learning approaches, the relationships between personal values and academic achievement, including the effect of factors such as gender and academic discipline, has been carried out by scholars. As the literature notes, the achievement motive and achievement goal are different in their nature, but they both share a commonality in terms of the role that individuals’ values may play as their underlying antecedents [44]. The argument is further confirmed citing Kaplan and Maehr [45], and they contend that individuals’ achievement goals are associated with their values. Similarly, values are considered desirable goals and individuals work hard to pursue them. Hence it is justifiable to say that in the academic setting students personal values or their personal goals substantially influence the academic achievement of the students.

Among the several research studies made to study the impact of values on academic achievements, Bala [46] discusses the values and adjustment problem of high achievers and low achievers based on a sample of 100 students from two senior secondary schools. There, the researcher has considered values in terms of theoretical, economic, aesthetic, social, political and religious values and adjustments related to social, health and emotional, school and home values. Achieving one of the specific objectives to determine the nature of the values of High and Low achievers, it arrives at several conclusions: (a) Higher achievers are more theoretical and social in comparison to low achievers and they have a dominant interest in knowledge, learning and believe more in kindness, charity and love; (b) High achievers and low achievers are similar as far as religious value is concerned; (c) Low achievers are more economic in comparison to high achievers. They believe more in materialistic life than high achievers; (d) High achievers are more political in their approach in comparison to low achievers; (e) Low achievers are superior on the aesthetic value in comparison to high achievers.

There has been little research to study the effect of a school’s disciplinary climate on improving students’ learning and academic achievement. However, the available past and present research support the view that student learning is immediately affected by the nature of the school’s disciplinary climate [4] as it controls students’ conduct by restricting the engagement in misbehaviour during school time and, thus, enhance student learning.

According to Ma and Willms [47], research findings based on a sample of grade 8 students in the US, the two most important disciplinary factors that affect academic achievement pertain to whether students were concerned about class disruptions, the proportion of students who talked to a school counsellor or teacher about disciplinary matters and the effect of the teacher–student relationship. As they revealed, with respect to the effects of indiscipline on academic achievement, the disciplinary measure that had the strongest relationship to academic achievement pertains mainly to classroom disruption. Additionally, they say that the effect of behaviour concern, which is a more traditional indicator of disciplinary climate, was negatively related to academic success. As they have found that students’ indiscipline has a significant detrimental effect on their academic achievement, to improve academic achievement from the perspective of a disciplinary climate, providing an orderly classroom environment has been suggested as a remedy.

Research conducted at the individual level has consistently shown a correlation between low cognitive ability, poor academic performances, learning disabilities, delinquency and particularly the relationship between academic performances and discipline [47]. In schools where advantaged students are concentrated, there will be fewer discipline problems and higher achievement levels as they completely target academic success rather than other issues, whereas schools serving disadvantaged students will have even worse discipline problems and lower levels of academic achievement. Ma and Willms [47] support that claim with Hawkins and Lishner [48], who have framed the relationship between academic performance and discipline as a circular process. School misconduct in the early elementary grades, combined with low ability or learning disabilities, are antecedents of poor academic performance in the late grades; poor academic performance in the late elementary grades leads to a low commitment to educational activities, disaffection toward school and an association with delinquent peers. These factors lead to dropping out or to delinquent behaviour. Value education is another concerned faculty that is gaining much concern in education. The results of a study on students attending character education and some of which did not have shown that the scores of those who underwent character education were higher than the scores of others [49]. As a whole, according to these authors, schools’ or any other learning community’s disciplinary climate acknowledges that better-behaved students generally are higher academic achievers. On that basis, as highlighted in the aforementioned discussion, if personal values are considered as abstract ideals that guide people’s behaviour, then there should be a correlation between delinquent behavioural patterns, cognitive ability level, academic performance and the personal values of an individual.

Liem et al. [44] examined the relationships between values, achievement motives, achievement goals and academic achievement among Indonesian high school students. There, in terms of the relationships between values and achievement motives, findings indicate that security and conformity values are positive predictors of the social-oriented achievement motive; self-direction is a positive predictor of the individual-oriented achievement motive, whereas hedonism is a negative predictor of both achievement motive orientations. There is also evidence for the direct effects of values on academic achievement. How personal values influenced students’ learning approaches and in turn, how they related to students’ achievement has been examined several times, and they have resulted in more or less similar results, as in Liem et al. [44]. Accordingly, Wilding and Andrew [43], based on their study cohort behaviour, have observed that those with less interest in wealth and status life goals produced better academic results. In other words, the successful students would seem to apply themselves more (or more effectively) to the immediate task rather than wider ambitions. Hence, they concluded the two variables associated with better performance were a self-reported achieving approach to learning, reflecting good organization and a systematic programme of study and a lower emphasis on wealth and status achievement in life. Furthermore, they stress that Biggs’ achievment approach to learning has consistently been shown to be positively related to academic performance, but neither the surface approach nor the deep approach has shown any such consistent relation. In contrast to that, the results of a study on a sample of university students by Tarabashkina and Lietz [10] showed that specific combinations of values were related to each learning approach and their relationship with the academic achievement of students over three years. In general, certain consistencies of these relationships have been observed throughout the study period. The deep and achieving learning approaches were associated with higher achievement, whereas students who displayed more characteristics of the surface learning approach had lower academic performance. Through statistical analysis, they built up the positive and negative relationships between personal values and learning approach: (a) Achieving learning approach—self-direction, achievement and hedonism; (b) Deep learning approach—self direction and hedonism; (c) Surface learning approach—conformity and self-direction. As they found, if the deep and achieving learning approaches were associated with higher achievement, then it can be assumed that self-direction, achievement and hedonism values are consistently associated with academic achievements, affecting them negatively and/or positively.

Similarly, the research findings of the study on learning approaches of successful students done using freshman students ranked in the top one percent portion in a university placement exam (2013) in Ankara by Beyaztaş & Senemoğlu [50] were supported with the similar research literature and has shown that students can enhance their level of success by increased use of the deep learning approach and decreased use of the surface approach. Furthermore, references made in Watkins’s [51] meta-analysis of 60 studies addressing learning approaches and academic achievement found a negative relationship between academic achievement and surface learning approaches in 28 studies, a positive relationship between academic achievement and deep learning approaches in 37 studies and a positive relationship between academic achievement and strategic learning approach in 32 studies. Additionally, in a study by Senemoğlu [52] a positive and meaningful relationship was found between Turkish and American students’ perceived level of success and learning approaches. This study reported that students who perceived themselves to be successful tended to adopt deep and strategic learning approaches, whereas students who thought they were less successful used surface learning approaches in both countries. According to the outcomes of the above-mentioned research studies, any consistent assumptions cannot be made about the correlation between the effectiveness of the learning approaches and students’ academic achievements or about how learning approaches influence academic performance. As emerged in the previous research literature, students’ learning behaviour along with personal values may change according to the circumstances and, in turn, it makes a direct effect on the students’ academic achievement.

### 4.3. Objective 3: To Investigate the Importance of Developing Individual’s Personal Values as a Part of Their Academic Life

Education is a combined process in which the advancement of knowledge, development of skills and the acquisition of beliefs and habits progress from an earlier age. Education providers, especially schools, play an important role in helping young people to develop and manage their physical, social and emotional well-being, and to live and work with others in different contexts. Specifically, they are partly responsible for enlightening an individual in both personal and professional areas. In that sense, personal value development is given a prominent place in most of academic interventions since they are considered as the concepts of beliefs that guide behaviours, attitudes and social norms. Education is naturally and inevitably directly related to a person’s goals and values [53]. The objective of developing an individual’s personal values as a part of academic life has been discussed, mainly concerning the theme of value education in many of the studies. In general value, education occupies an impressive place in contemporary society and school education is the most influential means of developing an individual and the schools are meeting places of value and are also full of values [54].

Values education itself has been defined simply as a purposive attempt to teach what is good or bad. As Iscan and Senemoglu [49] define it, values education is an open initiative aimed to provide instruction in values, value development or value actualization. According to the definition underpinning the Value Education Study, Australia [55], ‘Values education’ is broader and refers to any explicit and/or implicit school-based activity to promote student understanding and knowledge of values and to inculcate the skills and dispositions of students so they can enact particular values as individuals and as members of the wider community. Beena [56] says that value education given at schools is much concerned with striving for personal wholeness as well as generating a responsible attitude towards others and an understanding of wrong and right behaviour. For Thornberg and Oguz [57], all kinds of activities in schools in which students learn or develop values and morality are often referred to as values education. It seems that through the value education at school, children are encouraged to explore the powers of good and bad while unconsciously setting appropriate limits to behaviour. In relation to the Schwartz theory of personal values, the school value education promotes the values (benevolence, universalism, tradition, conformity, security) that primarily regulate how one relates socially to others and affects their interests. Security and universalism values are boundary values primarily concerned with others’ interests, but their goals also regulate the pursuit of their own interests [19]. Particularly, schools being sites for ethical practices, it seems that they focus much on social value development rather than personal development. According to Kunduroglu & Babadogan [53], that may be because the values students get with values education affect firstly their families and circle of friends, then their acquaintances and at the end, all the community.

As Thornberg and Oguz [57] emphasize, referring to several studies, value education is accomplished in two distinct ways such as explicit values education (schools’ official curriculum of what and how to teach values and morality, including teachers’ explicit intentions and practices of values education and implicit values education (associated with a hidden curriculum and implicit values, embedded in school and classroom practices). Bergmark [54] also mentions that schools are full of implicit and explicit values which shape school leaders’, teachers’ and students’ perceptions and actions. Furthermore, Thornberg and Oguz [57] mention two general approaches to values education as described in the literature. The first is the Traditional Approach: adult transmission of the morals of society through character education, direct teaching, exhortation, and the use of rewards and punishments. The aim is to teach and discipline students to develop good character and virtues (being honest, hardworking, obeying legitimate authority, kind, patriotic and responsible) and to conform to the dominant values, legitimate rules and the authority of society. In contrast, the Progressive or Constructivist Approach emphasises children’s active construction of moral meaning and development of a personal commitment to principles of fairness and concern for the welfare of others through processes of social interaction and moral discourse. Reasoning and explanations, deliberative discussion about moral dilemmas and participation in decision-making processes are viewed as typical methods for this approach. The aim is to promote moral autonomy, rational thinking, moral reasoning skills and democratic values and competence among the students.

Values education has always been a part of the school curriculum in many countries aiming to inculcate religious beliefs, moral values, duties and social responsibilities as the social values are of crucial importance for an individual’s life [53]. Therefore, the personal value development of students is important as it is beneficial for the individual in academic, professional and social life. Academic development achieved without personal value development is worthless because individuals who are not disciplined find it difficult to survive in the long run of professional and social life. They lack positive qualities such as punctuality, flexibility, the willingness to learn, a friendly nature, an eagerness to help others, sharing and caring and many more. In addition, they do not believe in themselves and others and lack self-confidence, self-efficacy and self-courage, which are considered the main components of personal development. Obviously, educating people on an only cognitive level is incomplete and not functional [53]. Henceforth, academic growth must be supplemented with personal value development to strengthen the individual to fit in the competitive society and do away with negative behavioural traits. That gives the sense that better personalities yield positive results in academics, social and professional life.

The research study by Iscan and Senemoglu [49] on the effectiveness of values education curriculum for fourth graders to equip students with the values of “universalism” and “benevolence” on students’ value-related cognitive behaviours, affective characteristics and performances has resulted in important findings. The experimental group of the study has shown higher values-related cognitive behaviour acquisition level and used more expressions reflecting values in the interviews during and after the implementation of the program. Additionally, the experimental group has displayed a larger number of positive value-related behaviours during the study than the control group. In parallel to the particular study, Iscan and Senemoglu [49] highlight the the importance of value-based educational interventions. As they revealed, exposing students to such experiences may make them aware of moral issues, establish empathy with others and understand their moral values, decreasing bullying and violence. Furthermore, they have made students more tolerant, polite, compassionate and forgiving, and [58] it has led to positive changes in students’ respect and responsibility levels along with a decrease in unacceptable behaviour. A similar study on “Values Education Program Integrated with the 4th Grade Science and Technology Course’’ [53] has revealed that at the end of the 6-week intervention period, students in the experimental group improved their perspective on the values, being more open-minded, unbiased and scientific. In addition, they have interrogated values concepts and developed positive behaviours for the relevant values.

As a whole, it proves that value education is an essential component in the general teaching-learning procedure since it highly encourages positive personal quality development and value gain which in turn benefit the whole community, society and the world.

### 4.4. Objective 4: To Investigate How One’s Personal Value Shape the Learning Community around That Person and Vice-Versa

Definitions for learning communities that have been given by a variety of journals, top universities and educational experts indicate a common set of characteristics. Considering them all together, a learning community can be defined as the same groups of students taking the same subjects or studying in the same class together. In addition, they see and meet each other frequently, share the same learning experiences, work across boundaries, spend a considerable amount of time together and engage in common academic activities in two or more classes as a specific unit. Additionally, they hold common goals, characterize collaboration, peer review and relationship building.

Sometimes the learning community can be the whole class or a group of students. Otherwise, it can be the whole learning institution: a school, university or any other institution where the individuals of the community develop their intellectual and professional skills and abilities while improving socioethical values. In addition, they work collaboratively as a single unit for achieving a set of common academic goals, sharing and bearing all kinds of similarities and differences [58]. In a more formal sense, according to the literary evidence, developing and implementing an intentional learning community (LC) has emerged as a popular method for improving the quality of the undergraduate experience at a range of higher educational institutions. Learning communities have a long history in higher education, dating from the 1920s when Alexander Meiklejohn introduced the “Experimental College” at the University of Wisconsin [59].

It is known that, from early ages, pupils are greatly influenced by their peers [21], and this has been empirically studied. Zhao and Kuh [58] state that students who actively participate in various out-of-class activities are more likely to connect with an affinity group of peers, which is important for student retention, success and personal development. Peer communities sometimes encourage and sometimes discourage value development as the students encountered different learning activities. Ma and Willms [47] view peer relationships are associated with delinquency in early adolescence. So, the potential role of peers as an influential factor on others in the process of values formation at the schools has been studied several times. In this respect, the study of Garnier and Stein [60] confirms that peer groups in which people interact and share norms and goals are another significant matter that affects the personal values of an individual. One important source of values is that of a ‘pivotal’ person: a person observed as displaying values that would produce advantageous benefits for the observer [4]. In a learning community, there is a possibility of a friend or friends becoming a pivotal person or persons other than the teacher or the instructor. Hence, it is evident that learning communities trigger personal value development through peers, their behaviours and attitudes and all the personal attributes.

To address the above features through the teaching and learning process, different approaches have been taken by the educational practitioners to figure out the best way to teach their students, and many have failed. However, some have succeeded and are still on the ground with alterations and developments. Among them, the cooperative learning strategy has continued to be developed and used by the teachers at all levels. Hence, by exposing students to collaborative or cooperative learning experience, they are encouraged to work together with colleagues to achieve common targets. As the word sense, it is not just group work but a very dynamic strategy [61] that provides room for students to experience different personalities, to promote social interaction, to identify sociocultural dynamics, to transfer ideas, and to develop group leadership skills among students. Cooperative learning is a teaching practice that breaks students into groups of three to four, with each student having a particular role within the group [61]. However, collaborative learning goes beyond working together, and it inspires self-management, self-monitoring and self-directed earning while developing a core skill required for employment [62]. In that sense, when comparing the intended outcomes of collaborative and cooperative learning approaches with the Schwartz’s [19] categorisation of values, they enhance values such as self-direction, achievement, benevolence and universalism.

Zhao and Kuh [58] refer to several studies, and according to them, most learning communities incorporate active and collaborative learning activities and promote involvement in complementary academic and social activities that extend beyond the classroom. Such approaches are linked with such positive behaviours such as increased academic effort and outcomes such as promoting openness to diversity, social tolerance and personal and interpersonal development. In parallel to that, Stassen [59] points out the results of the empirical studies collectively and show that “living-learning communities have a significant positive effect on several student outcomes, including: student gains in autonomy and independence, intellectual dispositions and orientations, and generalized personal development and socialization”. Stassen [59] mentions that students in learning communities show greater institutional commitment, greater intellectual development and opportunities to analyse and integrate ideas, greater tolerance for difference and appreciation for pluralism and demonstrate higher persistence and academic performance as measured by college grade point average.

Taken together, by taking classes together and/or engaging in peer-to-peer learning as a learning community, students get to know each other better, learn from each other and support each other. Along with that, students experience more social relationships. A connected learning environment increases the potential for academic success while creating more opportunities for students to adapt themselves to the individual needs of each other, to adjust their schedules and to work with diverse groups since learning groups are a mixture of different intellectual abilities, academic interests and goals and learning styles. Then again, social relationships established as a result of learning communities will continue through the end of the academic experience and will last even after promoting social harmony. As explained in Schwartz’s [1], benevolence values provide an internalized motivational base for voluntarily promoting the welfare of others. Equally, conformity values promote prosocial behaviour to avoid negative outcomes for oneself. Hence, both benevolence and conformity values motivate the same helpful act of promoting cooperative and supportive social relations, separately or together. As discussed above the learning communities also directly or indirectly enrich the development of values such as benevolence and conformity in learners, since they support the natural integration of academic life with social life providing opportunities to interact with a variety of individuals. In turn, the learning community will be benefited or disturbed by the certain characteristics of the personal values held by the individual.

## 5. Discussion & Conclusions

Based on the above literature on the themes of personal values and related directions, it is clear that there is no universally accepted definition for personal values. However, despite the diversity and gaps in the definitions, values and personal values have been viewed basically as the concepts or beliefs which are depicted through behavioural patterns, selections and personal goals. Furthermore, intrinsic and extrinsic factors including family, social and economic background, neighbourhood, religion and education have been identified as the influential factors on value formation and development. Their effect on the life of a person alternate according to the circumstances. Jardim et al. [63] identified this nature of values as the two main functions: as a motivator (materialist or humanitarian law) or as guidance (personal, social or central). Furthermore, based on the different attributes of values and priorities given to them in different contexts, they have been defined, named and grouped in various ways with more or fewer similarities to each other. However, both Schwartz [19] and Jardim [63] explained the similarities of values and value systems. As they state values have a basic universal structure and character which make them to be believed as the judgment of truths. The emphasis given to values in many areas has resulted in a number of theories and frameworks, and they have been used as the theoretical grounds to evaluate the research outcomes. According to the search results of this particular study revealed that Schwatrz theory of personal values has been frequently used in many of the recent education-based research studies in comparison to the other theories.

The study of personal values can provide greater insight into the entirety of human behaviour. Therefore, it has been studied concerning a variety of disciplines including education. Although there are a limited number of educational studies dealing with values, attempting to explore the relationship between personal values and learning approaches, personal values and academic achievement, influence of one’s personal values on learning community and vice versa and value education are important trends that emerged in educational research. Those studies mainly focused on identifying students’ preferred learning approaches at different stages of academic life and underlying values that are likely to influence the preference. In addition, the positive and negative behaviours of the underlying values with the learning approaches over time and the changes were aimed at. When concerned with the learning approaches that are found frequently in studies, the deep, surface, achieving and strategic approaches are prominent. According to Wilding and Andrews [43], the two main approaches to studying are the deep approach and the surface approach, as distinguished by several researchers. In addition, an achieving or strategic approach employs either deep or surface strategies, depending on the demands of the task. Contrastingly, Matthews et al. [3] and Lietz and Matthews [27] cite Biggs [29], and he has specified three distinct approaches to learning, namely, The Surface, The Deep and The Achieving approaches to learning. The classification of Biggs’ [29] learning approaches appeared in many of the studies related to personal values, learning approaches and academic achievements. Research by Matthews et al. and Lietz et al. [3,27,37] based on personal values and their effect on students’ preference for learning approaches have revealed similar relationships and their changes over time, mainly related to the underlying values along with the other factors. In fact, revealing the correlation among value, learning approach and academic achievement is extremely important for educational practices. However, as they conclude, there is no consistency in those changes, and it has been further revealed that one learning approach is influenced by several value attributes. In general, deep and strategic learning approaches are found to be positively related to the academic achievement of successful students, whereas the surface learning approach is reported with less successful students. Self-direction and achievement values were identified as the most influential in students’ success through the above approaches. Collectively, the above study results offer potential insights that may be useful when designing new academic courses or in any teaching-learning intervention. Furthermore, though personal values are not the sole determinant of educational or career choice, the correct understanding of values is useful in addressing the arising needs and issues in any discipline. Especially to address a wide range of issues relating to schooling and any educational outcomes such as academic achievement, retention, participation, dropping out, discipline and career selection.

With regard to today’s transforming society, value education has identified a crucially important requirement. Both the cognitive and affective domains of a child need to be developed through education. Kunduroglu and Babadogan [53] stressed that the purpose of education is to furnish students with affective behaviours. Mainly, schools and other educational institutions are the places where students continue their value education process, which begins at home. One of the objectives of values education in schools is to develop a healthy, consistent and balanced personality in students [16]. In that sense, formal educational interventions are better focused on enhancing the values that children have already started to develop and help children to reflect, understand and implement their own values accordingly. At this point, direct or indirect inclusion of themes such as moral, religious, civic, democratic, national, personal and social goals and issues in the school curricula has been stressed as important. Furthermore, the need of treating value education as a high priority in terms of ensuring the continuity of society and cultural transmission at a personal level also highlighted in many studies. The effectiveness of curricula including value education has been studied several times, and the results revealed the robust links between value education, student disciplinary conduct and academic achievements. Additionally, the consideration given to the respective roles of formal and informal education, learning communities, peers, parents and other institutions and agencies in making sense of values and forming personal values is emphasized in much of the value-education-based research.

Another concept that emerged as important in the dimension of personal values is its close relationship with the learning community and vice versa. The peer group influence on shaping academic behaviour and personal behaviour have long been studied by scholars over different perspectives. Concerning that, many researchers have focused on cooperative/collaborative learning interventions as the means of establishing social relationships and value development.

In general, when analysing the contents of research studies, it was notable that research related to personal values and learning approaches have been the major focus of many scholars in comparison to the other directions. A few studies found online databases discussing the relationship between personal values and academic achievement. Study reports directly focusing on the correlation of personal values and learning community and vice versa and the importance of personal values as a part of academic life are found lacking in online databases. Methodologically, it was found that many of the studies tend to apply mixed method designs and only a few have taken qualitative and quantitative research as their main research method. Other than that, literature-based reports are also available as useful academic resources. In the data collection process, questionnaires and interviews were found as the most commonly used instruments.

The discussion of personal values includes many distinct dimensions and can be approached through numerous perspectives: education, personal and social life, professional world, culture, political, religion and so on. It is realized that focusing only on a part of it cannot result in a holistic study of the concept but still it would be important to understand the depth of the concept. Depending on online resource availability and the time period set for the selection of resources for the current review may have resulted in the exclusion of some valuable research outcomes and directions. However, the comparative analysis based on available literature would probably shed light on the variety of interpretations, findings and research tendencies.

Finally, as the research literature reveals, the insight gained through the results of value-related studies facilitate the clear identification of the role of value in personal life and partly as a deciding factor of academic life. If one is not clear of his or her own values, then he/she is not clear with aims and is ineffective in controlling their life. Hence, further investigation on value-related topics over the wide range of its interrelated dimensions would give a more holistic and profound view of the role of personal values in education.

## 6. Recommendations

Based on the above discussion, it is apparent that still there is much room for future research studies on the theme of personal values since they affect all the avenues of human life, individually or in common as a group or a community. Conversely, several factors influence personal values and their changes. Therefore, a detailed further examination of the complex interplay of factors influencing personal values and how personal values influence an individual and in common to the whole human community seems to be valuable.

According to the analyses presented in this article, it is implied that the topic of personal values is very much important in the field of education to identify students’ behaviours, life goals and expectations, learning styles and how these change over time. Furthermore, increased attention is given to value education since values are considered as essential social or soft skills that one must acquire and practice in the 21st century world. Therefore, education, regardless of the level of junior, secondary, tertiary or professional, should aim at making human life better not only through professional or economic enhancement but also through social, moral and spiritual strengthening. At present, schools and other educational providers have adopted several co-curricular programmes that uplift values in students, such as peer support systems, community service projects and student action teams. These interventions provide students with opportunities to develop a sense of responsibility, empathy, unity, appreciation of others and their views, lifestyles and cultures and work with others to resolve the problems. These programmes have been recorded with notable achievements. This is a common feature of almost all the educational contexts that ensure values are incorporated into teaching programmes across the key learning areas to develop students’ civic and social skills. Thus, there is a need for a realistic and balanced curriculum in which the programs that inspire the value acquisition and internalisation of socially beneficial skills and behaviours are emphasized. In addition, the integration of such features into the disciplines in the curriculum is also important. Along with that, research studies to evaluate the strengths and weaknesses and the positive and negative aspects of such programmes need to be continued. According to the general and most practiced procedure, during or at the end of the academic experience, cognitive behaviours are always tested, but testing effective behaviours is always neglected. Hence, it is a noteworthy point to mention the importance of assessing processes for the progress of value development in students.

Finally, the current study based on the available literature has shown that students probably tend to adjust their approaches to a specific learning strategy due to several factors: learning environment, subject area, expectations, curriculum, teacher and teaching style, origin and cultural context, gender, religion, etc. Furthermore, there is no significant pattern of selecting learning approaches such as deep, surface or achieving, etc., at different levels of the context of learning. Therefore, deep study into how learning approaches are changed, on what basis and what the most influential motives for such alterations are will be beneficial to understanding students’ learning behaviours. Hence, research studies further investigating such dimensions would probably useful and needed at present and in future.

## Figures and Tables

**Table 1:** Values and the motivational goals—the Schwartz theory of personal values.

Value	Motivational Goals
**Conformity**	Restraint of actions, inclinations and impulses likely to upset or harm others and violate social expectations or norms. (obedient, self-discipline, politeness, honouring parents and elders, loyal, responsible)
**Tradition**	Respect, commitment and acceptance of the customs and ideas that one’s culture or religion provides. (moderate, spiritual life, respect for tradition, humble, devout)
**Benevolence**	Preserving and enhancing the welfare of those with whom one is in frequent personal contact (helpful, honest, forgiving, responsible, loyal, true friendship, mature love)
**Self-Direction**	Independent thought and action—choosing, creating, exploring. (creativity, freedom, choosing own goals, curious, independent, self-respect, intelligent, privacy)
**Stimulation**	Excitement, novelty and challenge in life. (A varied life, an exciting life, daring)
**Hedonism**	Pleasure or sensuous gratification for oneself. (pleasure, enjoying life, self-indulgent)
**Achievement**	Personal success through demonstrating competence according to social standards. (ambitious, successful, apable, influential, intelligent, self-respect, social recognition)
**Power**	Social status and prestige, control or dominance over people and resources (authority, wealth, social power)
**Security**	Safety, harmony and stability of society, of relationships and of self. (clean, Family and national security, social order, reciprocation of favours)
**Universalism**	Understanding, appreciation, tolerence and protection for the welfare of all people and for nature. (broadminded, social justice, equality, peace, unity with nature, wisdom, protecting the environment)

**Table 2:** Motivations and strategies in student approaches to learning.

Approach	Motive	Strategy
**SA: Surface**	Surface Motivation (SM) is instrumental: to meet requirements minimally; a balance between working too hard and failing	Surface Strategy (SS) is reproductive: to limit the target to bare essentials and reproduce through rote learning
**DA: Deep**	Deep Motivation (DM) is intrinsic: study to actualize interest in what is being learned; to develop competence	Deep Strategy (DS) is meaningful: read widely, interrelating with previous relevant knowledge
**AA: Achieving**	in academic subjects Achieving Motivation (AM) is based on competition and ego-enhancement: to obtain the highest grades, whether or not material is interesting	Achieving Strategy (AS) is based on organising time and working space; to follow up suggestions; behave like a ‘model’ student

Note. MNNote. Matthews et al. (2007) [3] following Biggs [29] and Murray-Harvey [31].

**Table 3:** Inclusion and exclusion criteria.

Type of Criterian	Creiteria	Inclusion	Exclusion
Type of publication	Journal articles	*	
	Conference papers	*	
	Reports	*	
	Dissertations		*
	Books		*
Access	Online	*	
	Paper		*
Publication period	2000–2020	*	
Place of publication	World wide	*	
Types of study	Emphirical studies	*	
	Theoritical studies	*	
Research methods	Quantitaive	*	
	Qualitative	*	
	Mixed	*	

## Data Availability

The data presented in this study are available on request from the corresponding author. The data are not publicly available due to privacy.
